# ABCC6 Involvement in Cerebral Small Vessel Disease: Potential Mechanisms and Associations

**DOI:** 10.3390/genes16070728

**Published:** 2025-06-23

**Authors:** Marialuisa Zedde, Rosario Pascarella

**Affiliations:** 1 Neurology Unit, Stroke Unit, Azienda Unità Sanitaria Locale-IRCCS di Reggio Emilia, Viale Risorgimento 80, 42123 Reggio Emilia, Italy; 2 Neuroradiology Unit, Ospedale Santa Maria della Misericordia, AULSS 5 Polesana, 45100 Rovigo, Italy; rosario.pascarella@aulss5.veneto.it

**Keywords:** small vessel disease, SVD, ABCC6, stroke, lacunar, calcifications

## Abstract

ABCC6, a key regulator in ectopic calcification, plays a crucial role in mineralization through the modulation of extracellular purinergic pathways and production of inorganic pyrophosphate (PPi), which inhibits calcification. Inherited deficiencies in ABCC6 lead to pseudoxanthoma elasticum (PXE) and related conditions, characterized by calcification in various tissues, particularly affecting the skin, eyes, and cardiovascular system. Although PXE does not directly impact the nervous system, secondary neurological issues arise from cerebrovascular complications, increasing the risk of strokes linked to arterial blockages resembling atherosclerosis. This review investigates the connection between ABCC6 mutations and cerebral small vessel disease (SVD), expanding the understanding of PXE and related phenotypes. Mutations in ABCC6, identified as causing PXE, contribute to systemic metabolic dysfunction, with significant implications for cerebrovascular health. An association between ABCC6 mutations and cerebral SVD has been suggested in various studies, particularly in populations with distinct genetic backgrounds. Emerging evidence indicates that pathogenic mutations increase the risk of ischemic strokes, with both homozygous and heterozygous carriers showing susceptibility. Mechanistically, ABCC6 deficiency is implicated in dyslipidemia and atherosclerosis, further exacerbating cerebrovascular risks. Increased arterial pulsatility, linked to carotid siphon calcification, may also contribute to microvascular damage and subsequent brain injury. Understanding these mechanisms is vital for developing targeted diagnostic and therapeutic strategies for managing cerebrovascular risks in PXE patients. This review emphasizes the need for comprehensive genetic screening and the consideration of traditional vascular risk factors in patient management, highlighting the complex interplay between genetic mutations and environmental influences affecting cerebrovascular health. Future research should focus on longitudinal studies to elucidate the causal pathways linking arterial calcification, pulsatility, and brain damage in PXE.

## 1. Introduction

Adenosine triphosphate (ATP)-binding cassette sub-family C member 6 (ABCC6) belongs to the ATP-binding cassette superfamily [1] and plays a critical role in regulating ectopic calcification [2,3]. This protein influences mineralization by modulating the extracellular purinergic pathway through several essential enzymes that generate inorganic pyrophosphate (PPi) and inhibit its degradation [4,5,6]. Notably, PPi serves as a strong inhibitor of ectopic calcification [7]. The activity of ABCC6 contributes to around 60% of plasma PPi levels in both humans and mice [8,9]. The liver and kidneys exhibit the highest levels of ABCC6 expression, although this protein is also present in various other cell types [10,11]. A deficiency in ABCC6 due to genetic inheritance can result in calcification of the dermis, eyes, and cardiovascular system in individuals with pseudoxanthoma elasticum (PXE) [3], and is implicated in some instances of generalized arterial calcification of infancy (GACI) [12]. Additionally, it may be a contributing factor to PXE-like symptoms in patients with β-thalassemia [13,14]. Besides arterial calcifications, PXE does not have a direct impact on the nervous system; however, secondary neurological issues due to cerebrovascular limitations are well established, although present in a variable proportion of patients. Older patients with PXE are at a higher risk for developing cerebrovascular events, which often stem from the gradual narrowing and blockage of one or several cerebral arteries [15]. The angiographic characteristics of PXE are similar to those seen in severe atherosclerosis, exhibiting features such as arterial stenosis, tortuosity, and occlusion, particularly affecting the internal carotid or vertebral arteries. Carotid artery stenosis, accompanied by significant collateral circulation, bears a resemblance to moyamoya syndrome in angiographic findings [16,17]. Findings similar to those of fibromuscular dysplasia have also been reported [18]. There is a slightly elevated incidence of intracranial arterial aneurysms among individuals with PXE, likely due to their abnormal elastic lamina [19]. These aneurysms frequently occur in the intracranial segment of the internal carotid artery [20,21,22], while aneurysms in other intracranial vessels are rarer. A case was noted where a woman experienced subarachnoid hemorrhage and spinal cord dysfunction due to an aneurysm in the anterior spinal artery [23]. Both intracerebral and subarachnoid bleeding have been observed in PXE patients without the presence of an aneurysm [24,25].

In recent years, the association of some pathogenic mutations of ABCC6 with a microvascular cerebral lesion pattern, or with a neuroimaging pattern consistent with a cerebral Small Vessel Disease (SVD), has been reported, albeit occasionally [25,26,27,28]. Furthermore, some similar reports, both in the European and Asian populations, concern one of the manifestations of SVD, namely lacunar ischemic stroke. This association may be due to different mechanisms and can be either direct or mediated by the co-presence of some vascular risk factors.

The aim of this review is to explore the association between ABCC6 mutations and SVD and to illustrate the mechanisms of this, substantially expanding the range of PXE and PXE-like phenotypes.

## 2. ABCC6 and PXE

PXE is an autosomal recessive genetic disorder caused by mutations in the ABCC6 gene on chromosome 16 16p13.1, identified in 2000 [3,29,30,31,32,33], which codes for a transmembrane transporter protein of the ATP binding ABC family. The prevalence of PXE is around 1 in 25,000 people, but some individuals with milder phenotypes probably remain undiagnosed. This gene consists of 31 exons and encodes a 1503-amino-acid protein (molecular weight: 165 kDa). The literature encompasses 48 ABC (“adenosine triphosphate (ATP) binding cassette”) genes, categorized into seven subfamilies (A to G). The ABCC subfamily includes 12 genes, among which are ABCC6 and ABCC7 (CFTR, linked to cystic fibrosis), along with a pseudogene (ABCC13). Due to structural similarities, the protein encoded by ABCC6 is classified within the multidrug resistance protein subfamily, known for exporting organic ions from external sources (e.g., cancer drug metabolites) [34]. In fact, older studies may refer to ABCC6 as MRP6. The ABCC4, ABCC5, ABCC11, and ABCC12 proteins possess two membrane-spanning domains and two nucleotide-binding domains. SUR1 and SUR2 (from ABCC8 and ABCC9) also exhibit four domains, while ABCC1, ABCC2, ABCC3, ABCC6, and ABCC10 have an additional N-terminal domain. A three-dimensional model of ABCC6 has been proposed based on homology, with high-resolution structures of other ABC proteins [35], but its accuracy remains uncertain. ABCC6 gene expression is tissue-specific [36], with indications that a primate-specific sequence (+629/+688) in the first intron might play a role [37]. The binding of hepatocyte nuclear factor 4 α (HNF4α) to a conserved promoter region (−209/−145) may explain the gene’s predominant expression in the liver [38]. The transport mechanism of endogenous or exogenous substrates by ABCC6 is poorly characterized. Despite its classification in the MRP family, the molecular pathways for drug or metabolite transport by ABCC6 remain undefined, suggesting it has limited involvement in clinical multidrug resistance. In vitro studies indicated that ABCC6-transfected cells did not show significant resistance to various chemotherapeutic agents [39]. In autosomal recessive disorders, heterozygous carriers of a mutation in one ABCC6 allele typically do not exhibit PXE symptoms [40,41]. However, some heterozygotes may display clinical and histopathological PXE characteristics [42,43,44]. The observation of mineralized skin in a woman with ABCC6 and GGCX mutations raised the possibility of a milder PXE form (OMIM #177850). It is plausible that undetected mutations could affect the other allele, maintaining the recessive inheritance pattern [45]. Additionally, heterozygotes for ABCC6 mutations may have an increased risk of cardiovascular calcification [46].

While PXE’s genetic basis is acknowledged, its pathophysiological mechanisms are not fully understood. Although ATP secretion from the liver is ABCC6-dependent, ATP is not transported by the protein. This ABCC6-dependent ATP secretion is a primary source of circulating pyrophosphate (PPi) [4,8]. In Abcc6 (−/−) mice, plasma PPi levels are about 40% of those in wild-type mice, and PXE patients show a low plasma PPi/Pi ratio [4,8]. Thus, PPi is proposed as a key circulating factor in PXE metabolic disorder [8,47]. Despite ABCC6 being primarily expressed in the liver, kidney, and intestine, damage to PXE occurs at distant sites. Two main hypotheses exist: the cell-based hypothesis, suggesting that dysfunctional ABCC6 at peripheral sites leads to ectopic mineralization [48], and a systemic metabolic hypothesis positing that insufficient circulating factors from the liver result in mineralization elsewhere. One variant of this hypothesis indicates that these circulating factors normally inhibit mineralization, and their absence leads to dystrophic calcification in tissues like skin, eyes, and arteries. Experimental evidence in Abcc6-deficient mice supports this metabolic hypothesis, as pairing these mice with wild-type mice halted connective tissue mineralization, likely due to the introduction of critical anti-mineralization factors from the wild-type circulation [49]. PPi remains a strong candidate for the anti-mineralization factor in PXE [8,47]. Elevated Pi levels have been implicated in calcification based on dietary experiments in Abcc6 (−/−) mice [47], yet PXE patients maintain normal parathyroid hormone levels, and a clinical trial of sevelamer hydrochloride showed no significant effects on calcification [50]. The results may have been influenced by excipient components. If Pi plays a role in PXE, it may be through a reduced PPi/Pi ratio [4,8]. Other potential players in PXE include the anti-mineralization proteins matrix Gla-protein (MGP) and fetuin-A, which were found to be moderately low in PXE patients and significantly low in chronic kidney disease (CKD) patients [51,52]. MGP knockout mice exhibit spontaneous arterial and cartilage calcification [53]. A CKD model showed low Abcc6 protein levels despite normal mRNA levels, suggesting post-transcriptional or post-translational deficiencies [54]. Additionally, it has been hypothesized that impaired vitamin K export from the liver decreases the γ-carboxylation of anti-mineralization proteins [55,56]. MGP is not carboxylated in PXE patients’ elastic fibers [57], and PXE-like calcification is observed in GGCX mutation patients [57]. However, failed supplementation trials in PXE mouse models challenge the vitamin K hypothesis [58,59,60]. Adenosine is another candidate circulating factor, given the parallels between PXE and “arterial calcification due to deficiency of CD73” (ACDC), where extracellular adenosine monophosphate cannot convert to adenosine [61,62]. Patients with ACDC and CD73-deficient mice develop dystrophic calcification [63,64], but this hypothesis is weakened by the lack of adenosine transport by ABCC6 in vitro [65]. Oxidative stress is also considered a potential factor in PXE, as some patients show biochemical signs of stress [66], and conditions like β-thalassemia or sickle cell anemia (characterized by elevated free radical levels) can present PXE-like symptoms [67,68,69,70]. Furthermore, oxidative stress may inhibit ABCC6 expression in human cell lines. One study reported abcc6 localization to mitochondria-associated membranes in mice [71], but other studies confirmed its primary basolateral membrane localization in human and mouse liver [72]. Gene expression analyses in wild-type, Abcc6-deficient, and Abcc6-transgenic mice indicated that impaired substrate export from hepatocytes alters the regulation of systemic anti-mineralization factors (the “hepatic intoxication” hypothesis). However, differences in gene expression were minor and not statistically significant after correction [73], with metabolic changes in the liver not reflected in plasma profiles [73]. Liver function remains intact in PXE patients. Most experimental data on PXE’s pathophysiology derives from Abcc6-deficient zebrafish [74,75] and mouse models [76,77,78,79,80]. In mice, all Abcc6 −/− models develop dystrophic mineralization, with calcium deposits in the skin, retina, and arteries mirroring human PXE. For instance, arterial calcium accumulation is significantly higher in Abcc6 −/− mice compared to wild-type [81]. One study highlighted the activation of the BMP2-SMAD-RUNX2 signaling pathway, critical for vascular calcification, in Abcc6-deficient mice [82].

The main molecular mechanisms involved in PXE manifestations are summarized in the following table (Table 1).

Different hypotheses were proposed for the factors pathologically involved with PXE, which are the main ABCC6 expression in the liver and the main PXE manifestations outside the liver, as follows: (I) the metabolic hypothesis (the decrease or loss of ABCC6 functionality, especially in the liver, may lead to a decrease in circulating factors in the bloodstream, whose role is to prevent the ectopic mineralization of soft tissues); (II) the PXE cell hypothesis (the absence of ABCC6 in PXE tissues alters cell proliferation and changes in the biosynthetic pathway affect cell interactions with the extracellular matrix); (III) the ATP release hypothesis (ABCC6 mediates the efflux of ATP into the extracellular milieu; ATP is hydrolyzed into AMP and pyrophosphate and this process prevents the mineralization of soft tissues).

Genotype–phenotype correlations are generally weak [83]. The nonsense mutation p. Arg1141* may predispose individuals to cardiovascular disease independent of hyperlipidemia [84,85], while the ABCC6 p. Arg1268Gln polymorphism [30,86] is associated with early onset of angioid streaks [87,88]. ABCC6 mutations have also been connected to generalized arterial calcification of infancy (GACI; OMIM 173335), which is linked to ENPP1 mutations that regulate bone mineralization [89]. GACI is often lethal in utero or within months of birth, with ENPP1 mutations found in most affected patients [90].

At present, there are no universally recognized international guidelines for the clinical and genetic diagnosis of pseudoxanthoma elasticum (PXE). In the past, before the identification of the ABCC6 gene’s involvement in PXE, diagnosis was based on three major and two minor criteria [91]. The major criteria were as follows: (i) the presence of distinctive skin manifestations characterized by yellow cobblestone lesions in flexural areas, (ii) specific histopathological features of lesional skin evaluated with elastic tissue or von Kossa stains, and (iii) ocular complications, including angioid streaks, peau d’orange lesions, or maculopathy in individuals aged over 20. The minor criteria encompassed histopathological characteristics of non-lesional skin and a family history of PXE among first-degree relatives. However, this classification often does not correlate well with molecular findings related to ABCC6 [92]. In 2010, a revised classification system was proposed [93] (see Table 2). This semi-standardized method includes the following: (i) a comprehensive skin examination conducted by a dermatologist or physician familiar with PXE, (ii) histological analysis using hematoxylin–eosin, Verhoeff–van Gieson (to assess elastin), and von Kossa (to detect calcium) staining of a biopsy from an affected lesion or, if necessary, from the lateral neck, and (iii) fundoscopy of both eyes by a skilled ophthalmologist to evaluate for peau d’orange, angioid streaks, macular degeneration, comets, and wing signs, with optional fluorescein or indocyanine green angiography and fundus autofluorescence for angioid streaks [93]. In clinical practice, the identification of characteristic yellow cobblestone skin lesions typically prompts screening for ABCC6 mutations.

As previously mentioned, testing for ABCC6 mutations is conducted in patients unless the clinical presentation is unequivocal. So far, over 2000 unique DNA sequence variants of the ABCC6 gene have been recorded, with the majority being missense mutations [94]. Among these variants, there are 76 classified as conflicting, 103 as benign, 549 as likely benign, 826 as variants of uncertain significance, 232 as likely pathogenic, and 333 as pathogenic mutations. Approximately 90% of patients diagnosed with clinical PXE have mutations in both alleles of the gene. Notably, the mutation profile varies across different ethnic groups [95]. For example, the p.Arg1141* (p.R1141X) mutation is commonly found in European populations [95] but is less prevalent in North American groups [2,3], and was completely absent in a study of 22 Chinese patients, who instead exhibited 15 previously unreported mutations [96]. The del23-29 mutation is particularly common in Northern Europe and the northern Mediterranean, while the p.Gly1321Ser mutation is frequently observed in North America but is rare in Europe [2,3]. The p.Arg1138Trp missense mutation may act as a genetic marker for individuals of French ancestry, being present in France and French-speaking Canada, whereas the 2542delG frameshift mutation is mainly found in Japanese patients [95]. In contrast, mutations such as p.Gln378* and p.Arg1339Cys appear to have a similar distribution globally, suggesting recurrent mutational events. Generally, disease-associated missense mutations tend to cluster at domain–domain interfaces, exhibiting a mutation rate that is 4.25 times higher [97]. Copy number variations in the ABCC6 pseudogenes, ABCC6Ψ1 and ABCC6Ψ2 [98,99], have been found more frequently in PXE patients than in controls, although the clinical relevance of these variations remains unclear [100,101]. Furthermore, non-pathogenic polymorphisms have also been identified; for instance, an individual homozygous for the ABCC6 p.Arg1268Gln polymorphism did not show any PXE symptoms, and the Gln1268 (Q1268) allele was present at a frequency of 0.19 among healthy controls [31].

However, the estimated prevalence of PXE, at between 1 in 25,000 and 1 in 50,000, suggests a significant number of heterozygous carriers in the general population [102], irrespective of whether the full clinical spectrum of PXE is observed, leading to diagnostic tests [93,103]. It is possible that a genetic diagnosis may be even wider than a diagnosis triggered by clinical issues, as defined in the first and revised diagnostic criteria [104]. A recent study analyzed a French cohort of PXE patients, aiming to identify genotype-phenotype correlations, specifically through a comprehensive molecular analysis of the ABCC6 gene [105]. The study involved 458 French PXE probands referred for molecular analysis and a modified Phenodex score was used to evaluate the severity of symptoms [103]. The genetic analysis included sequencing of the ABCC6 gene and its pseudogenes, focusing on identifying both known and novel variants. Among the 306 PXE patients analyzed, the majority were women (63.1%) and Caucasian (79.4%), with a median age of 43.5 years. Neurovascular manifestations, such as strokes, were noted in 10% of cases, while renal lithiasis was detected in 12.9% [106]. A total of 538 mutational events were identified, with a detection rate of 87.7%. This included 142 distinct variants, of which 66 were novel. The most common variants identified were the nonsense variant p.Arg1141Ter and the del23-29 deletion, which were particularly prevalent among Caucasian patients [86,103]. Among the 306 cases, 81.7% had two identifiable variants, with many patients showing a homozygous status for certain variants. Two families exhibited pseudodominant inheritance patterns, which confirmed the recessive nature of PXE. Most missense variants were located in intracellular domains of the ABCC6 protein. The study highlighted specific regions within the protein that were enriched with pathogenic variants, particularly in the nucleotide-binding fold regions [8]. The analysis of 220 cases with complete Phenodex scores showed that skin lesions were the most common manifestation, present in over 90% of cases with two variants. The severity of ocular and vascular manifestations was significantly associated with the presence of loss-of-function variants [93]. The severity of PXE was influenced by the number and type of variants. Cases with two loss-of-function variants exhibited more severe eye and vascular complications compared to those with missense variants. Ethnic background also played a role, with Caucasian patients experiencing more severe ocular complications than North African patients [30]. This study found a high detection rate of causative variants in the ABCC6 gene, similar to previous studies [103]. However, it also highlighted the need for complementary diagnostic methods, such as array comparative genomic hybridization, to improve variant detection. The findings confirmed that the most common variants were consistent with previous reports, while new correlations were identified regarding the ethnic background of patients. The phenotype variability observed in PXE suggests the influence of additional genetic and environmental modifiers. The study proposed modifications to the Phenodex score to include additional clinical features, such as nephrolithiasis and stroke, which are prevalent yet overlooked in the standard assessment.

## 3. Small Vessel Disease Phenotype and ABCC6 Mutations

Cerebral SVD accounts for approximately 25% of strokes, manifesting as lacunar strokes and deep intracerebral hemorrhages (ICH) [107,108], and serves as the primary pathology behind vascular cognitive impairment [109]. Cerebral SVD is characterized by various neuroimaging findings, such as lacunar infarctions (LIs), enlarged perivascular spaces (EPVS), microbleeds (MBs), and white matter hyperintensities (WMHs). It is a significant cause of dementia and gait disturbances [110]. While traditional risk factors include aging and hypertension, the pathogenesis of SVD is still not fully understood. In most instances, it is a sporadic age-related condition associated with hypertension and subsequent arteriosclerosis, although a minority of cases can be attributed to rare genetic variants [111]. More than 10 genes are implicated in monogenic forms of cerebral SVD, including NOTCH3 and HTRA1, which have been increasingly recognized in the recent literature [112]. Diagnosing monogenic cerebral SVD (mgCSVD) is often challenging due to the absence of distinct clinical features in some patients and the potential for the disease to occur without a family history. This necessitates genetic screening to identify cases of mgCSVD even when familial patterns are not evident. The most prevalent inherited form of SVD is cerebral autosomal dominant arteriopathy with subcortical infarcts and leukoencephalopathy (CADASIL), which is caused by NOTCH3 variants [113]. Recently, additional genes have been identified that can lead to similar phenotypes, including HTRA1, COL4A1, COL4A2, TREX1, GLA, and FOXC1 [114]. However, the prevalence of these variants in populations with presumed sporadic SVD remains unknown and several other variants of different genes which are not routinely searched for could be at least equally prevalent. As the understanding of monogenic SVD broadens, a gene-by-gene testing approach becomes less cost- and time-effective. High-throughput sequencing (HTS) panels utilizing next-generation sequencing technologies facilitate the simultaneous testing of multiple genes associated with a single disease phenotype in a more economical manner and are increasingly adopted in clinical settings. In a recent description [115], an HTS panel that includes 15 genes associated with the SVD phenotype was evaluated for its effectiveness in disease diagnosis and to ascertain the frequency of monogenic disease-causing variants in a well-defined population with MRI-confirmed younger-onset lacunar stroke. This investigation assesses both known disease-causing mutations and novel, potentially pathogenic variants. The gene panel was crafted to encompass seven genes established as causal for SVD (NOTCH3, HTRA1, FOXC1, COL4A1, COL4A2, TREX1, GLA), alongside eight genes linked to disorders that exhibit SVD-related phenotypes. These include familial cerebral amyloid angiopathy (APP, CST3, ITM2B), familial hemiplegic migraine (ATP1A2, CACNA1A, SCN1A), and connective tissue disorders (ABCC6, COL3A1). These conditions share clinical features with monogenic forms of SVD (for instance, lacunar stroke, MRI WMHs, dementia, migraine with aura, and encephalopathy) and may thus present similarly. In the above-mentioned study [115], the study population consisted of patients from the UK DNA Lacunar Stroke Study, collecting unrelated patients of European ancestry with MRI-confirmed lacunar stroke occurring at or before the age of 70 [116]. Interestingly, no ABCC6 mutations were identified in this cohort. The same finding was proposed by a more recent study analyzing a cohort of 257 patients with suspected familial cerebral SVD and 13,086 controls [117]. Besides these cohorts, single case reports and small case series have noted an association between ABCC6 mutations and SVD [26].

Conversely, in a Japanese study in patients with adult-onset SVD [118], more than 90% of monogenic cerebral SVD (mgCSVDs) were diagnosed by screening for NOTCH3, HTRA1, and ABCC6, concluding that the target sequences for these three genes could be used to efficiently diagnose mgCSVD in Japanese patients. The study focuses on the role of these genetic factors in Japanese patients, emphasizing the need for effective genetic screening. The study recruited patients with adult-onset severe CSVD characterized by significant WMHs. Group 1 included patients aged 55 or younger, while Group 2 consisted of those older than 55 with a family history of CSVD. Genetic testing was performed for NOTCH3 and HTRA1, followed by whole-exome sequencing (WES) for undiagnosed cases. The study also included measuring HTRA1 protease activity and assessing clinical and imaging features through statistical analyses. A total of 106 patients were initially recruited, with 75 in Group 1 and 31 in Group 2. The study identified various mutations: 30 patients had NOTCH3 mutations, 11 had HTRA1 mutations, and 6 had ABCC6 mutations. Notably, the total mutation frequency for NOTCH3, HTRA1, and ABCC6 was 94% in patients with mgCSVD. Significant differences were observed in family history, hypertension prevalence, and multiple LIs between monogenic and undetermined cases. In Group 1, 54.7% of patients had mgCSVD, while in Group 2, the frequency was 29.0%. Among the mgCSVD cases, mutations in NOTCH3 accounted for 56.1%, HTRA1 for 24.4%, and ABCC6 for 12.2%. The findings suggested that screening these three genes could efficiently diagnose mgCSVD in the Japanese population. The results showed that HTRA1 and ABCC6 mutations significantly contribute to severe cerebral SVD in Japanese patients. While CADASIL was prevalent, the study highlighted the role of HTRA1 and ABCC6, even in heterozygous states, as important genetic risk factors. The neuroradiological selection criteria for the study are a relevant issue. The study recruited patients with adult-onset severe symmetrical WMHs corresponding to Fazekas grade 3 and various conditions, like LIs or MBs, on MRI. Genetic testing identified CADASIL in 30 patients, with several novel mutations in the NOTCH3 gene noted. Additionally, mutations in HTRA1 were found in nine patients, with two being novel. Among the undiagnosed patients assessed through WES, several mutations in ABCC6 were identified, including one with a heterozygous mutation resulting in PXE. When the clinical features of mgCSVD patients were compared to those of undetermined patients, the former had a higher frequency of family history, LIs, and non-lobar MB distributions. Statistical analysis indicated that family history, hypertension, and multiple LIs were significant predictors for mgCSVD diagnosis. This study demonstrated that over 50% of patients with severe CSVD who developed symptoms before 55 years had identifiable genetic mutations, with CADASIL being the most prevalent. Notably, HTRA1 and ABCC6 mutations were significant contributors to mgCSVD cases. The results indicate that these mutations can occur even in heterozygotes, emphasizing the necessity of genetic testing in diagnosing severe CSVD in Japanese patients, regardless of family history. The high frequency of ABCC6 mutations in this cohort suggests a potential link between these genetic factors and severe CSVD. The findings advocate for targeted genetic testing strategies, focusing on NOTCH3, HTRA1, and ABCC6, to enhance diagnostic efficiency for mgCSVD in Japan. Unfortunately, similar information in different populations is not currently available.

**Figure 1:**
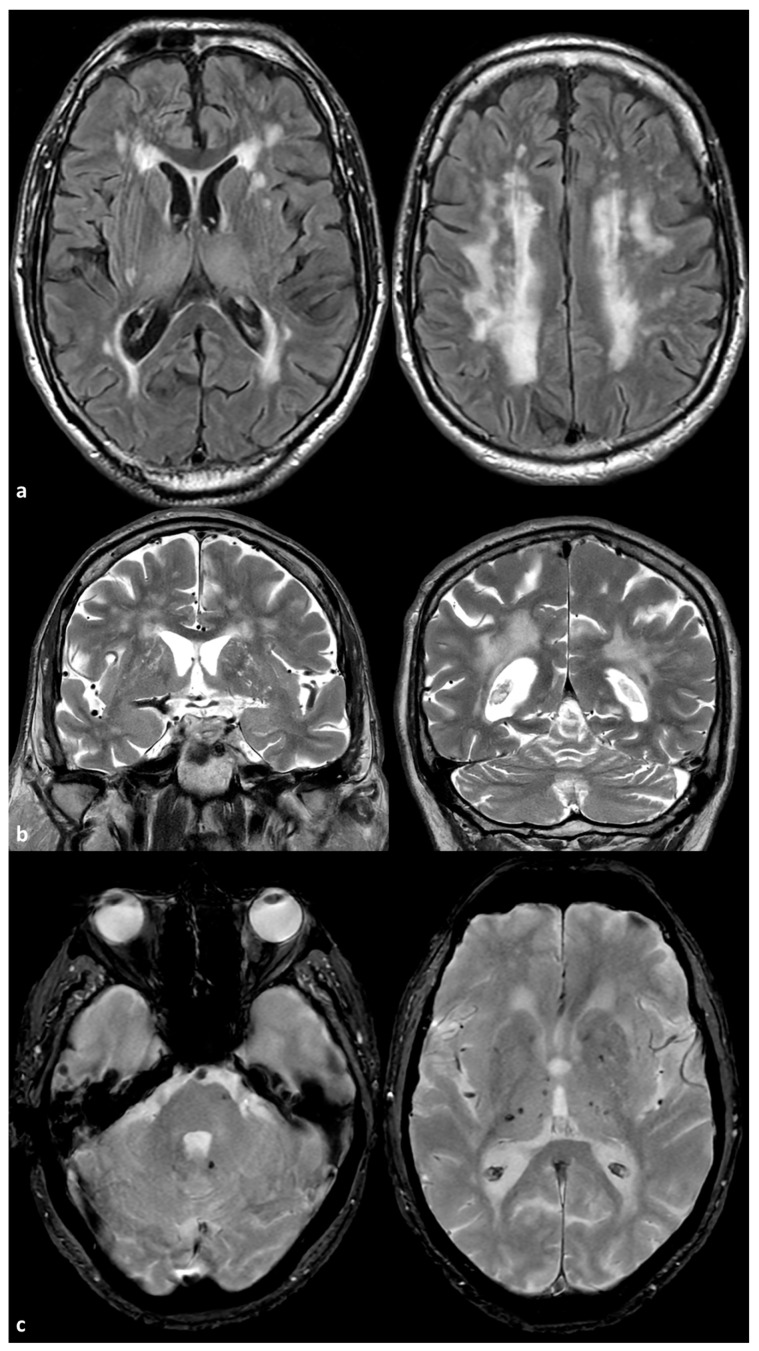
Brain Magnetic Resonance Imaging (MRI) of a 72-year-old man with a mutation ABCC6(NM_001351800.1):c.3840delG:(p.Lys1280AsnfsTer9) in heterozygosis. The patient has a very mild arterial hypertension and was sent to the neurological attention because of the occurrence of transient neurological deficit. In panel (**a**), some examples of axial Fluid Attenuated Inversion Recovery (FLAIR) images are provided at basal ganglia level (**left image**) and at centrum semiovale level (**right image**), showing symmetric, extensive, confluent white matter hyperintensities that are prevalent in the centrum semiovale. In panel (**b**), coronal T2W images show the enlarged perivasculae spaces in the basal ganglia on both sides. In panel (**c**), Gradient Recalled Echo (GRE) images show isolated pontine and deep, supratentorial, hypointense rounded signals similar to those observed for microbleeds.

**Figure 2:**
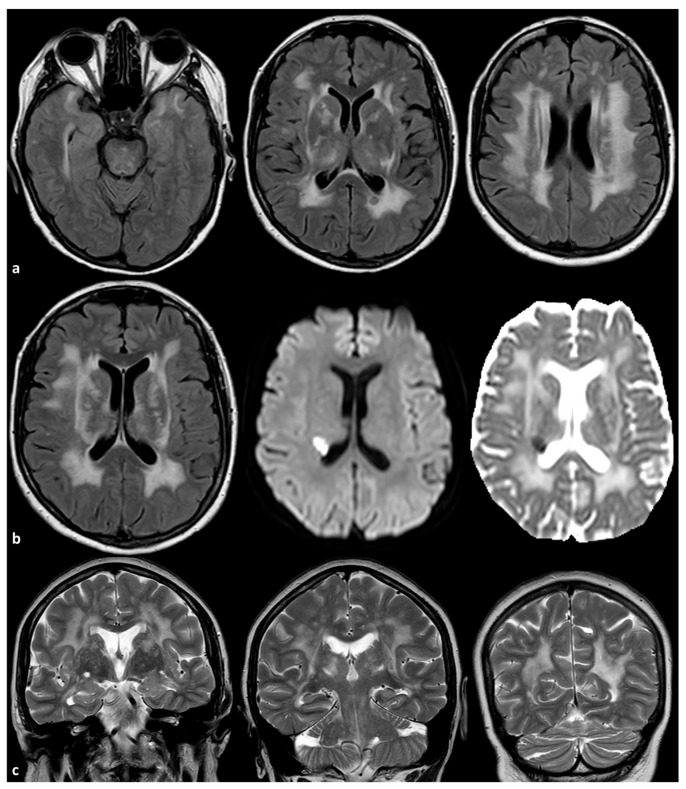
Brain MRI of a 56-year-old woman with a mutation ABCC6 (NM_001351800.1):c.3071G>A:p.(Arg1024Gln) in heterozygosis. The patient had no relevant vascular risk factors and no systemic signs of PXE. In panel (**a**), axial FLAIR images are provided, showing symmetric, extensive, confluent white matter hyperintensities involving the anterior temporal pole, the external and extrema capsula, the thalamus and the centrum semiovale on both sides (the other known genes responsible for SVD were tested and no mutations were identified). In panel (**b**), axial FLAIR, axial Diffusion Weighted Imaging and axial Apparent Diffusion Coefficient map images show an acute subcortical ischemia in the corona radiata close to the occipital pole of the right lateral ventricle. In panel (**c**), coronal T2W images show the enlarged perivascular spaces in the basal ganglia on both sides.

Two examples of severe SVD in patients with ABCC6 mutations are illustrated in Figure 1 and Figure 2.

## 4. Mechanisms Linking ABCC6 Mutations to Small Vessel Disease

At least one cohort study raised noted pathogenic mutations of ABCC6 and the risk of stroke [119]. That study highlighted the increased incidence of ischemic stroke in patients with PXE, including patients with monoallelic and biallelic pathogenic ABCC6 variants. Interestingly, the authors found that heterozygous ABCC6 variants significantly increase the risk of ischemic stroke. The study involved two main cohorts: a multigenerational family with a history of ischemic cerebrovascular events and an independent cohort of 424 ischemic stroke patients compared to 250 healthy controls. A genetic analysis of the ABCC6 gene was performed to identify pathogenic variants, including the recurrent multi-exon deletion. Clinical evaluations included a complete cardiovascular examination and neuroimaging to classify stroke types. In a large multigenerational family, the pathogenic ABCC6 variant (p.[Arg1314Gln]) was segregated alongside cerebrovascular and cardiovascular diseases. This family exhibited an autosomal dominant pattern of inheritance, with multiple family members affected by ischemic events at a relatively young age. The case–control study revealed that pathogenic ABCC6 variants were found in 16 out of 424 ischemic stroke patients (3.8%) compared to 2 out of 250 controls (0.8%), resulting in an odds ratio of 4.9 (*p* = 0.036). This suggests that carriers of pathogenic ABCC6 variants have a significantly higher risk of developing ischemic stroke. Additionally, Abcc6-deficient mice show Bmp (bone morphogenetic protein) and Tgfβ (transforming growth factor β) dysfunction in induced acute cardiac ischemia [120,121,122]. Similar BMP and TGFβ dysfunction was observed by us in PXE patients [123]. The immunostaining of brain tissues from Abcc6-deficient mice indicated dysregulation of Bmp and Tgfβ signaling pathways, suggesting a pro-ischemic state that may contribute to the increased risk of ischemic events in patients with ABCC6 mutations. The results advocate for the inclusion of molecular analyses of the ABCC6 gene in the diagnostic workup for patients with cryptogenic ischemic stroke, as early identification could influence management strategies. Interestingly, in the case–control study, only one out of sixteen patients had an SVD-related stroke pattern, and Del23-29 (Exon 23-29) was found in ABCC6 gene. In fact, the stroke phenotypes linked to pathogenic ABCC6 variants are varied, primarily including large vessel disease, followed by cardioembolic stroke and SVD, aligning with previous findings in PXE patients [45,46]. This heterogeneity complicates the identification of a specific subgroup of ischemic stroke patients predisposed to these variants; however, a negative family history should not dismiss the possibility of genetic influence. In an independent stroke patient cohort, none exhibited the significant intracerebral vascular calcifications or anatomical malformations previously noted in PXE patients [124]. In addition, the increased cardiovascular risk in heterozygous carriers of pathogenic ABCC6 variants may be due to other members of the ABC transporter superfamily that are involved in brain disease [125,126,127]. After stroke, increased ABCB1 expression was observed, negatively affecting neuroprotective agents [127]. In contrast, variants in ABCA1 linked to a reduced risk of ischemic stroke were identified [125]. Immunostaining on targets within the Bmp and Tgfβ signaling pathways (Bmp4, Bmp9, Alk2, Eng) in the Abcc6-deficient brain found the upregulation of Bmp4, a protein with confirmed pro-apoptotic properties [128]. Additionally, the downregulation of Alk2, which also leads to increased apoptosis, was noted [129]. Furthermore, the upregulation of Eng was observed; this pathway is crucial for VEGF-induced angiogenesis in ischemic conditions [129,130]. Overall, this suggests a pro-ischemic state is present in the brain tissue of Abcc6-deficient mice, potentially explaining the higher risk of ischemic stroke in patients with pathogenic ABCC6 variants. The primary limitations of this study include the relatively small sample size and the heterogeneity of the patient group suffering from various stroke subtypes, complicating the demonstration of minor differences between groups, as reflected in the wide confidence interval of the odds ratio. Nevertheless, a significantly higher presence of pathogenic ABCC6 variants in the stroke patient cohort compared to healthy age- and sex-matched controls was demonstrated, independently of other risk factors. Given that even minor triggers could lead to acute ischemic events in these patients, stricter management of other cerebrovascular and cardiovascular risk factors, such as tobacco use, obesity, and hypercholesterolemia, is advisable to minimize the risk of ischemic stroke or cardiovascular issues.

Another potential mechanism linking ABCC6 with stroke comes from animal studies suggesting there is an indirect pathway involving the classic vascular risk factors [131]. In fact, the study reveals that ABCC6 deficiency contributes significantly to dyslipidemia and atherosclerosis in both mice and humans. Mice lacking the ABCC6 gene exhibited altered lipoprotein profiles, specifically decreased HDL cholesterol and increased LDL levels, which corresponded with enhanced atherosclerotic plaque formation [8,132]. The absence of ABCC6 was linked to decreased cholesterol efflux from macrophages and increased systemic inflammation, indicated by elevated levels of pro-inflammatory cytokines such as IL-6 and CCL-2 [85,133]. These factors likely exacerbate the atherosclerotic phenotype observed in both Abcc6-deficient mice and PXE patients [2,134,135]. Despite the noted dyslipidemia and atherosclerosis, the study found that ABCC6 deficiency did not significantly affect vascular calcification associated with atherosclerosis. The vascular mineralization appeared to be a consequence of atherosclerotic plaque rather than a direct effect of ABCC6 genotype [136]. In a cohort of PXE patients, significant reductions in HDL levels were observed, mirroring the findings in the ABCC6-deficient mice. However, total cholesterol levels did not differ significantly from healthy controls, suggesting a complex interplay between ABCC6 deficiency and lipid metabolism [2,3,137,138]. Overall, the findings establish that ABCC6 plays a crucial role in modulating plasma lipoproteins and the hapolinsufficient development of atherosclerosis, with implications for understanding the cardiovascular risks associated with ABCC6 mutations. The study emphasizes that further investigation into cellular and molecular mechanisms is necessary to fully elucidate the pathways involved in these processes. These putative mechanisms are summarized in Table 3.

Other potentially relevant factors were provided by a neuroradiological study [139]. This study investigated the neuroradiological findings associated with PXE, particularly focusing on increased intracranial arterial pulsatility and microvascular brain damage, using Magnetic Resonance Imaging (MRI) and Computed Tomography (CT)-based techniques (Table 4).

Patients with PXE demonstrated significantly higher pulsatility indexes (1.05) compared to controls (0.94) (*p* = 0.02), indicating increased arterial flow pulsatility in the intracranial arteries. This elevated pulsatility is likely a consequence of carotid siphon calcification, which contributes to the high prevalence of cerebrovascular disease observed in PXE patients [140,141]. The findings suggest that increased pulsatility may lead to microvascular damage in the brain, contributing to structural changes such as lower gray matter volumes (597 mL vs. 632 mL in controls, *p* < 0.01), more white matter lesions (2.6 mL vs. 1.1 mL, *p* = 0.05), and a higher number of lacunar infarctions (64 vs. 8, *p* = 0.04) [124,142,143]. These results align with the pulsatility hypothesis, which posits that increased pulsatility damages brain tissue by leading to stress on the microvasculature, including the role of vascular calcifications [144]. The presence of carotid siphon calcification is associated with increased flow pulsatility, which in turn correlates with microvascular brain damage, including infarctions and white matter lesions [145,146].

## 5. Potential Implications for Future Studies

These findings underscore the need for further research to elucidate the mechanisms linking arterial calcification and increased pulsatility to brain damage in PXE. Longitudinal studies that monitor changes in pulsatility and calcification over time could provide insights into the causal pathways involved in cerebrovascular events among PXE patients [147]. Understanding the relationship between pulsatility and small-vessel disease could also inform therapeutic strategies aimed at reducing vascular risk in this population, potentially focusing on interventions that modify arterial stiffness or pulsatility [8]. Additionally, the study highlights the importance of evaluating traditional vascular risk factors in the context of PXE. A prevalence of hypertension and hypercholesterolemia was noted in the patient cohort, with 44% of PXE patients exhibiting hypertension and 86% having hypercholesterolemia [148,149]. These factors may exacerbate microvascular damage and warrant consideration in the management of patients with PXE. The interplay between genetic factors like ABCC6 mutations and environmental factors such as lifestyle and comorbidities may present a multifaceted challenge in understanding and addressing cerebrovascular risk in these patients [150,151].

Another less-explored issue in the ABCC6 literature is the association between biomarkers of extracellular matrix degradation, such as matrix metalloproteinases (MMPs) and their tissue inhibitors, and the progression of vascular diseases. Elevated levels of some of these biomarkers have been linked to increased vascular inflammation and remodeling, which are critical factors in conditions like atherosclerosis and hypertension and could be enhanced by the decrease or loss of function of the ABCC6 gene product. Table 5 summarizes some of these biomarkers and their potential role in vascular diseases.

From a personal perspective, integrating these biomarkers into clinical practice could enhance risk stratification and therapeutic monitoring, ultimately improving patient outcomes in vascular disease management.

## 6. Conclusions

ABCC6 is a pivotal regulator of ectopic calcification, primarily through its influence on the extracellular purinergic pathway and the modulation of PPi levels. Its deficiency is implicated in conditions such as PXE, underscoring the importance of this gene in mineralization processes. Patients with ABCC6 mutations exhibit an increased risk of cerebrovascular events, including ischemic strokes. The mechanisms linking these mutations to cerebrovascular complications involve dyslipidemia, atherosclerosis, and potentially altered hemodynamics, such as increased arterial pulsatility.

There is a pressing need for standardized genetic screening protocols to identify ABCC6 mutations in patients presenting with PXE and cerebrovascular manifestations. The current diagnostic criteria should be refined to incorporate genetic testing as a routine part of evaluation, especially for individuals with unexplained vascular conditions.

The clinical manifestations of ABCC6 mutations show significant variability, influenced by genetic background and environmental factors. This variability suggests the presence of additional genetic modifiers and highlights the need for personalized approaches to diagnosis and management.

Future research should involve longitudinal studies to track changes in cerebrovascular health in PXE patients over time. These studies could help establish causal relationships between ABCC6 mutations, arterial calcification, and the development of cerebrovascular complications. Investigating the broader genetic landscape surrounding ABCC6, including potential interactions with other genes implicated in vascular health, will provide deeper insights into the mechanisms underlying PXE and associated phenotypes. High-throughput sequencing technologies could facilitate this exploration. Detailed mechanistic studies are warranted to clarify how ABCC6 deficiency leads to dyslipidemia and atherosclerosis. Understanding these pathways could inform therapeutic strategies aimed at mitigating cerebrovascular risks in affected individuals. Employing advanced neuroimaging techniques to assess microvascular damage and arterial pulsatility in PXE patients could provide critical insights into the relationship between vascular changes and neurological outcomes, enhancing our understanding of the disease’s impact on the nervous system.

Finally, clinical trials targeting vascular risk factors in PXE patients, such as hypertension and hypercholesterolemia, should be conducted. These trials can help determine the efficacy of various interventions in reducing cerebrovascular events in this population. By addressing these research issues, we can improve our understanding of the complex interplay between ABCC6 mutations, ectopic calcification, and cerebrovascular health, ultimately leading to enhanced patient care and management strategies.

## Figures and Tables

**Table 1:** Main molecular mechanisms of ABCC6’s role in PXE manifestations.

Molecular Mechanism	Description
ABCC6 function loss	Decreased functionality of ABCC6 affects the transport of critical substrates, leading to metabolic disruptions.
ATP secretion	ABCC6-dependent ATP secretion from the liver is impaired; ATP is not directly transported by ABCC6.
Low Pyrophosphate (PPi) Levels	ABCC6 deficiency results in low circulating PPi levels, which are crucial for preventing ectopic mineralization.
Increased Inorganic Phosphate (Pi)	Elevated Pi levels may promote calcification; the low PPi/Pi ratio is implicated in PXE’s pathophysiology.
Ectopic Mineralization	Dysfunctional ABCC6 leads to mineralization in soft tissues (skin, eyes, arteries) due to the lack of inhibitory factors.
Altered Expression of Anti-mineralization Proteins	Impaired export of proteins like matrix Gla-protein (MGP) and fetuin-A, which normally inhibit mineralization.
Oxidative Stress	Increased oxidative stress may contribute to cellular damage and exacerbate PXE manifestations.
Dysregulation of Signaling Pathways	Activation of pathways such as BMP2-SMAD-RUNX2 associated with vascular calcification in the absence of ABCC6.

**Table 2:** New diagnostic criteria for PXE [93].

Major Diagnostic Criteria	
1. Skin	
a.	Yellowish papules and/or plaques on the lateral side of the neck and/or flexural areas of the body; or
b.	Increase in morphologically altered elastin with fragmentation, clumping, and calcification of elastic fibers in a skin biopsy from clinically affected skin.
2. Eye	
a.	Peau d’orange of the retina; or
b.	One or more angioid streaks (ASs), each at least as long as one disk diameter. If uncertain, fluorescein or indocyanine green angiography of the fundus is necessary for confirmation.
3. Genetics	
a.	A pathogenic mutation of both alleles of the ABCC6 gene; or
b.	A first-degree relative (parent, sibling, child) who independently meets the diagnostic criteria for definitive PXE.
**Minor Diagnostic Criteria**	
1. Eye	
a.	One angioid streak shorter than one disk diameter; or
b.	One or more ‘comets’ in the retina; or
c.	One or more ‘wing signs’ in the retina.
2. Genetics	
a.	A pathogenic mutation of one allele of the ABCC6 gene.
**Requirements for the Diagnosis of PXE**	
a. Definitive Diagnosis	Presence of two (or more) major criteria not belonging to the same (skin, eye, genetic) category.
b. Probable Diagnosis	Presence of two major eye criteria or two major skin criteria, or one major criterion and one or more minor criteria not belonging to the same category as the major criterion.
c. Possible Diagnosis	Presence of a single major criterion, or one or more minor criteria.

**Table 3:** Main molecular mechanisms underlying ABCC6’s role in SVD.

Mechanism	Description
Dysfunction of BMP and TGFβ Signaling	Dysregulation of bone morphogenetic protein (BMP) and transforming growth factor β (TGFβ) pathways in ABCC6-deficient models suggests a pro-ischemic state.
Pro-apoptotic Factors	Upregulation of Bmp4 and downregulation of Alk2 observed in brain tissue of Abcc6-deficient mice, leading to increased apoptosis.
Increased Cardiovascular Risk	Pathogenic ABCC6 variants correlate with increased cardiovascular risk; the mechanisms may involve other ABC transporters.
Dyslipidemia and Atherosclerosis	ABCC6 deficiency linked to altered lipoprotein profiles, decreased HDL, and increased LDL, contributing to atherosclerosis.
Systemic Inflammation	Increased pro-inflammatory cytokines (e.g., IL-6, CCL-2) in ABCC6-deficient models, exacerbating atherosclerotic phenotype.
Lipid Metabolism	ABCC6 deficiency modulates plasma lipoproteins; significant reductions in HDL levels observed in PXE patients.
Indirect Pathways	ABCC6 deficiency may contribute to classic vascular risk factors, influencing the development of ischemic strokes.

**Table 4:** Neuroradiological protocol employed in the above reported study [139].

Technique	Details
3T MRI Protocol	Patients underwent a standardized MR imaging protocol using a 3T MRI scanner. This high-field strength allows for better resolution and detail in imaging. - 2D Phase-Contrast MRI: This technique was specifically used to measure blood flow pulsatility in the intracranial arteries, including the internal carotid artery (ICA) and middle cerebral artery (MCA). The phase-contrast method provides time-resolved measurements of blood flow velocity, allowing for the calculation of the pulsatility index (PI). - 3D T1-Weighted Imaging: T1-weighted images were utilized to evaluate brain volumes, including gray matter (GM), white matter (WM), and cerebrospinal fluid (CSF). - 3D T2-Weighted FLAIR Imaging: Fluid-attenuated inversion recovery (FLAIR) sequences were employed to assess white matter lesions (WML) and identify any infarctions in the brain. This technique is particularly useful for highlighting lesions in the presence of CSF.
CT	- CT Scans for Carotid Siphon Calcification: Patients underwent CT imaging to visualize and quantify carotid siphon calcification. This involved unenhanced, thin-section CT scans, which were analyzed for calcification mass scores based on the area and density of arterial wall lesions. - Calcification Mass Score: An in-house-developed software tool was used to quantify the calcification mass score, allowing for objective assessment and comparison between patients with PXE and controls.
Image Processing	Custom MATLAB scripts were employed to analyze the 2D phase-contrast acquisitions, creating regions of interest (ROIs) for blood flow measurements and correcting potential phase wraps in the velocity maps.
Segmentation	Automatic segmentation of brain volumes and WML was performed using the Computational Anatomy Toolbox and the Lesion Segmentation Tool within SPM12 software. This automated approach allowed for the precise quantification of brain structures and lesions.

**Table 5:** Main extracellular matrix degradation biomarkers and their role in vascular diseases [152,153,154,155,156].

Biomarkers	Description
Matrix Metalloproteinases (MMPs)	- MMP-1: Involved in collagen degradation, linked to plaque instability in atherosclerosis. - MMP-2: Associated with vascular remodeling and fibrotic processes. - MMP-9: Elevated in various vascular diseases and correlated with inflammation and plaque rupture.
Tissue Inhibitors of Metalloproteinases (TIMPs)	- TIMP-1: Regulates MMP activity and is implicated in vascular remodeling; altered levels may indicate disease progression. - TIMP-2: May serve as a marker for vascular injury and fibrosis.
Collagen Degradation Products	- C-terminal telopeptide of type I collagen (CTX): Reflects collagen turnover and is associated with vascular stiffness and atherosclerosis. - N-terminal propeptide of type III collagen (PIIINP): Indicates extracellular matrix remodeling and has been linked to heart disease and vascular complications.
Elastin Degradation Products	Desmosine and Isodesmosine: Markers of elastin degradation, associated with vascular elasticity and stiffness.
Fibronectin and its Fragments	Elevated levels can indicate matrix remodeling and are associated with cardiovascular risk

## Data Availability

No new data were created.
